# The Great War: XI. Personal Cleanliness

**Published:** 1918-07-27

**Authors:** 


### THE GREAT WAR.

### XI. PERSONAL CLEANLINESS.

### II. Vermin.

"La Chasse."

One of the most striking phenomena encountered on a tour through the trenches is the good soldier's daily hunt through his underclothing—it constitutes a very valuable aid in the preventive campaign; the daily bag varying from four to twenty. When the bag exceeds the latter number there is generally consequent skin eruption, or "trench fever," necessitating retirement to hospital.

The De-lousing of Underclothing.

The dirty underclothing which is taken from the men at the baths is packed in sacks and dispatched at once to the Divisional or Corps Foden steam disinfectors, previously described, for disinfection. Ten hours are required for the de-lousing of the underclothing of a battalion, each load requiring one hour, including twenty minutes actual exposure to steam under a pressure of 5 lb.; this kills both lice and eggs infallibly. Occasional failures are due to neglect to keep up the fires.

The available Foden disinfectors are insufficient for the disinfection of blankets in addition to the underclothing, so they are augmented by Clayton sulphur disinfectors and by improvised steam disinfectors of the Serbian barrel type.

The Clayton Sulphur Disinfector.

The Clayton process is an absolutely reliable destructor of lice and eggs provided it is effectually carried out. It consists of a stove for the burning of sulphur candles and an engine driven by hand, petrol, or electricity, which delivers the resulting sulphur dioxide gas through a hose pipe by means of a rotary fan.

An hermetically sealable chamber is required, 30 ft. by 20 ft. and 10 ft. high (3,000 cubic feet), fitted with racks, on each side of a central passage, for the reception of sliding bars containing the articles for disinfection. These are placed at such a distance from each other as will allow free access of gas. Sulphur burnt in a closed chamber will exhaust the oxygen when it has produced 3 per cent. of sulphur dioxide, exposure to which does not kill lice, hence the necessity for the apparatus.

The original instructions for 30 minutes' exposure to 5 per cent. vapour are insufficient; protracted and carefully conducted experiments have proved that three hours' exposure to 7 per cent. vapour are necessary to kill eggs.

This degree of concentration is capable of maintenance in 3,000 cubic feet by one engine worked by electric or petrol power.

The Serbian Barrel.

The Serbian barrel type of disinfector is very common in the smaller units. It is a useful adjunct to the Divisional installations. In principle it resembles a potato steamer. It consists of a barrel, covered by another of larger calibre, into which steam is conducted from some sort of boiler below. Its capacity is ten blankets or equivalent underclothing at a time, and an exposure of one hour is necessary to kill eggs.

The Laundry.

After disinfection, the dirty underclothing is dispatched in clean sacks to the laundry. The weekly washing arrangements of our Army in the earlier days of the war were primitive and uncertain, and depended largely on personal effort and opportunity. Gradually, however, improvements were introduced leading up finally to the establishment of Divisional and Corps laundries.

These commenced modestly with manual labour and improvised material, progressing slowly and cautiously to the ideal metropolitan type fitted with every mechanical contrivance, and capable of dealing with as many as 80,000 to 100,000 sets per week.

This super-laundry, however, is exceptional; the ordinary Divisional laundry is not fully provided with machinery, partly because it is immobile, and partly as a question of policy, for these laundries provide a living for many poor women who have lost everything in their retreat from their homes in the lost territories.

Divisional laundries are established in disused breweries, factories, or school buildings. The washing and ironing is done by women, and the drying and collecting by men; socks are washed by machinery.

One hundred women are employed, each one washing thirty pieces daily. They work in pairs at fifty tubs, which are made by cutting margarine casks into two halves. Each woman is provided with a washing board (1 ft. by 3 ft.), a scrubbing brush, two-thirds of a bar of soap and a wooden mat, and the tubs are raised to a convenient height on stools.

The hours of work are from 8 to 12 a.m. and 2 to 6 p.m., and their pay three francs per diem.

The wet articles are lightly wrung out by the women, and the wringing is completed in four machines worked by men and capable of wringing 3,000 pieces daily; they are afterwards dried outdoors if the weather is suitable, at a drying ground 100 feet square provided with 1,000 yards of drying lines; if indoor drying is required, forty braziers are kept going by day and night in drying rooms. The weather is of economic importance, for the allowance of fuel, three-quarters of a ton of coal, and a similar quantity of coke is the maximum daily allowance for heating the water and for all other purposes.

When dry, every article is repaired by a staff of fifteen women whose wages are two and a half francs per diem, all mending materials being found by the

☞ Previous articles appeared Feb. 9, 23, March 9, 23, April 6, 20, May 4, 25, June 8, 22, and July 13, p. 321.

**The Great War**—(*continued*).

laundry. The shirts and pants are then ironed, thirty women being maintained for this purpose. Each is provided with two irons, and they work at tables 120 feet long and covered by Army blankets. After ironing the various articles are tied up into bundles of ten, collected by men, and taken to the clean linen store ready for issue.

The establishment of men authorised for a laundry of this size is two non-commissioned officers and twenty men for day work, and one non-commissioned officer and eight men for night work.

The washing of socks is done in two rotary machines, each worked by two women, and each turning out 500 pairs per diem.

There are few complaints of the weekly washing, which is distributed as a rule to the men at the baths after the weekly bath. Shrinking sometimes causes difficulty in fitting those who under this system may never wear the same shirt twice. The presence of dead " nits " on the shirts is also disconcerting. One can never be quite sure that the process of disinfection has not miscarried in any particular instance, and that the eggs are actually sterile. To meet this defect " combers " have recently been added to the establishment of laundries. They were at first provided with wire brushes, but they thinned the material and were otherwise inefficient, so that other plans were tried, leading up to the final selection of an ordinary clasp knife used as a scraper, and which has given entire satisfaction.

